# Space- and feature-based attention operate both independently and interactively within latent components of perceptual decision making

**DOI:** 10.1167/jov.23.1.12

**Published:** 2023-01-19

**Authors:** Guangsheng Liang, John E. Poquiz, Miranda Scolari

**Affiliations:** 1 Department of Psychological Sciences, Texas Tech University, Lubbock, TX, USA

**Keywords:** space-based attention, feature-based attention, perceptual decision making, drift diffusion model

## Abstract

Top-down visual attention filters undesired stimuli while selected information is afforded the lion's share of limited cognitive resources. Multiple selection mechanisms can be deployed simultaneously, but how unique influences of each combine to facilitate behavior remains unclear. Previously, we failed to observe an additive perceptual benefit when both space-based attention (SBA) and feature-based attention (FBA) were cued in a sparse display (Liang & Scolari, 2020): FBA was restricted to higher order decision-making processes when combined with a valid spatial cue, whereas SBA additionally facilitated target enhancement. Here, we introduced a series of design modifications across three experiments to elicit both attention mechanisms within signal enhancement while also investigating the impacts on decision making. First, we found that when highly reliable spatial and feature cues made unique contributions to search (experiment 1), or when each cue component was moderately reliable (experiments 2a and 2b), both mechanisms were deployed independently to resolve the target. However, the same manipulations produced interactive attention effects within other latent decision-making components that depended on the probability of the integrated cueing object. Time spent before evidence accumulation was reduced and responses were more conservative for the most likely pre-cue combination—even when it included an invalid component. These data indicate that selection mechanisms operate on sensory signals invariably in an independent manner, whereas a higher-order dependency occurs outside of signal enhancement.

## Introduction

Selective attention is routinely deployed to ensure desired visual input receives priority processing. In a goal-driven visual search task and with predictive pre-cued information, an observer may locate a target efficiently by deploying relevant selection mechanism(s). For example, relevant spatial information may be prioritized via space-based attention (SBA) such that visual representations in a selected location are enhanced, whereas input that falls outside the selected location is suppressed (Carrasco, 2006; Moran & Desimone, 1985; Posner, Snyder, & Davidson, 1980). Similarly, feature information (e.g. color, direction of motion, shape, et cetera) may be prioritized via feature-based attention (FBA): input that contains the attended feature is enhanced while unattended feature values are suppressed throughout the visual field (Motter, 1994; Rossi & Paradiso, 1995; Serences & Boynton, 2007).

Studies focused on SBA or FBA have greatly informed our understanding of each selection mechanism in isolation, both in terms of its impact on perception and its instantiation within the visual system. Researchers also keenly recognize, however, that in a real-world search, an observer is likely to have access to multiple forms of valid information regarding the target, leading to investigations into how multiple selection mechanisms interact when they are deployed simultaneously. Such lines of inquiry are especially important as they have the potential to provide greater insight into whether space and feature-based selection arise from a common or distinct mechanisms. Nonetheless, the findings of such studies thus far have been mixed. For example, single-unit electrophysiology studies demonstrated that SBA and FBA modulate sensory processing within area V4 independently (Hayden & Gallant, 2009; McAdams & Maunsell, 2000), suggesting unique mechanisms. In contrast, several behavioral studies have primarily relied on measures of reaction time (RT) to reveal significant interactions between space- and feature-based selection, suggesting a common mechanism (Bengson, Mangun, & Mazaheri, 2012; Kingstone, 1992).

In a set of experiments designed to unify these discrepant accounts, we recently argued that FBA and SBA likely operate on sensory signals in an independent manner, whereas higher-order mechanisms (i.e. attentional control and decision making) utilize space and feature information in a conjunctive, domain-general manner (Liang & Scolari, 2020). This conclusion was supported by a divergent pattern of results across performance measures (accuracy and RT) and drift-diffusion model outputs that are intended to capture underlying decision making components (Wagenmakers, Ratcliff, Gomez, & McKoon, 2008; Wagenmakers, Van Der Maas, & Grasman, 2007). When either location or color was pre-cued alone, we observed significant SBA and FBA effects of similar magnitudes. However, when both were pre-cued simultaneously, only SBA effects were observed in performance measures, whereas an interaction between selection mechanisms was observed within specific decision-making components. We reasoned that a common attentional control center within a network of frontoparietal regions, which coordinates goal-driven behavior, and is activated during space-, feature-, and object-based attention tasks (Corbetta & Shulman, 2002; Greenberg, Esterman, Wilson, Serences, & Yantis, 2010; Ibos & Freedman, 2016; Liu, Hospadaruk, Zhu, & Gardner, 2011; Serences, Schwarzbach, Courtney, Golay, & Yantis, 2004; Shomstein & Behrmann, 2006), may govern independent attentional modulations within sensory cortex.

Although this explanation may be compelling, on its own, the absent FBA effect we observed within accuracy and RT is equally consistent with both a dependent and an independent account of selective attention (Liang & Scolari, 2020). This leaves us with the task of trying to determine why FBA enhancement effects were absent. One possible reason for the reliance on one mechanism despite the dual cue is that both the valid location and valid color cues redundantly directed attention to a single item, rendering only one mechanism necessary to identify the target. In contrast, White, Rolfs, and Carrasco (2015) observed independent, additive FBA and SBA effects using a task in which each cue component made unique contributions to the search. This meant that each validly cued dimension reduced the search space of possible targets to a smaller subset, but a single target could only be isolated from all distractors using both valid cues in conjunction. The two studies additionally differed on cue validity, which could also contribute to differences in the pattern of results. The pre-cue used in Liang and Scolari was valid on both dimensions for 70% of trials, whereas this rate was much lower at 36% in White et al. Thus, participants could theoretically achieve a markedly higher accuracy rate in the former while using only one selection mechanism.

The latter possibility presupposes that attentional deployment is adjusted in accordance with cueing probabilities. Consistent with this, evidence shows that statistical regularities can influence how attention is distributed (Zhao, Al-Aidroos, & Turk-Browne, 2013), and that this can apply to locations in space and to specific feature values. In a recent electroencephalography (EEG) study, Arjona, Escudero, and Gómez (2016) manipulated the validity of a spatial pre-cue that indicated one of two possible locations of an upcoming monaural auditory stimulus with 50%, 68%, or 86% accuracy. The authors found that the size of the cueing effect within behavioral measures (RT and proportion of incorrect responses) scaled with the validity of the pre-cue, consistent with other reports (O'Bryan & Scolari, 2021; Riggio & Kirsner, 1997; Vossel, Geng, & Fink, 2014). A similar scaling effect has also been observed within FBA, albeit to a lesser extent (Huang, Xue, Wang, Hu, Bu, & Chen, 2018; Stilwell, Bahle, & Vecera, 2019). Furthermore, Arjona et al. detected scaling effects in ERP components related to perception, working memory updating, attentional reorientation, and sensory-motor preparation (P2a, P2p, P3a, and lateralized cognitive negative variation [CNV], respectively). Thus, such cueing regularities can exert influence at multiple levels of cognitive processing.

### Present study

The primary goal of the current study is to demonstrate that we can observe significant and independent SBA and FBA effects within sensory enhancement when both predictive spatial and feature information are provided in our paradigm, while also replicating the interaction in decision-making components. Notably, the valid pre-cues guide visual search to a relevant item without providing any information on the to-be-reported target element. Borrowing from White et al. (2015), we eliminated the redundancy of the cues such that target identification is only achieved by deploying FBA and SBA combinatorially on trials in which both cued dimensions are valid (Experiment 1). To preview the results, we found significant and independent SBA and FBA effects within measures of signal enhancement, and dependency in other components of perceptual decision making.

We next returned to a redundant cue while redistributing the frequencies attached to each dimension of the multidimensional cue (henceforth, cueing object) to investigate whether and how probability modulates the attention effects and their interactions within perceptual decision-making processes. First, we made both the location-only and color-only valid cues more likely than the wholly valid cue (Experiment 2a). Here, target identification can be achieved by utilizing one or the other mechanism on a given trial, but the likelihood of accurately attending the relevant item increases when both cues are used independently. Finally, we decreased the frequency of the location-only valid cue relative to the wholly valid and color-only valid cues (Experiment 2b) so that accurately attending the relevant item is more likely when using the feature cue.

By manipulating the probabilities attached to the color and location cues, we can investigate (1) whether and how attentional deployment is modulated by multiple, simultaneous probabilities across cueing dimensions, and (2) whether the probability of the whole cueing object is reflected in common or distinct manners across signal enhancement and other higher order components of decision making. Should we observe the latter pattern, this would provide further evidence that the intertwined processes utilize informative pre-cues in dissociable ways.

## Experiment 1

The goal of Experiment 1 was to modify the experimental design of Liang and Scolari (2020) Experiment 2 to elicit both SBA and FBA effects in a single task. Here, the location and color information indicated by an informative pre-cue each reflected at least one unique distractor (in addition to the target on valid trials), such that both cue types were necessary to completely isolate the target on wholly valid trials. We expected to find reliable, independent SBA and FBA effects in performance measures, while replicating our previously observed interactions within decision-making components.

### Methods

#### Participants

Because our goal was to replicate Liang and Scolari (2020), we set a target sample size to closely match that of Experiment 2 in the previous paper (*N* = 30). A total of 34 uninformed undergraduate students (9 men and 25 women) were recruited to participate through the Texas Tech Student Research Participation Program (SONA). All participants signed an informed consent form consistent with the Declaration of Helsinki and the Texas Tech Institutional Review Board. Each participant reported corrected-to-normal vision and had normal color vision as determined by an Ishihara color test (see Ishihara color test). Two participants were removed from analyses due to a programming error, and another participant was removed for having only one correct trial in one condition. Therefore, all analyses include the remaining 31 participants. All experiments lasted approximately 1 hour, for which participants received partial course credit.

#### Materials and stimuli

Participants viewed stimuli constructed from MATLAB (R2021b; MathWorks, Natick, MA, USA) and Psychtoolbox3 (Kleiner, Brainard, & Pelli, 2007) on a desktop computer running Windows 10. Stimuli were presented on a standard color gaming monitor (BenQ XL2430T) with a 1920 × 1080-pixel resolution display and operating at a 100-Hz refresh rate. Right eye fixation was monitored throughout the experiment with an EyeLink 1000 Plus infrared eye tracking camera (SR Research, Ontario, Canada) positioned in front of the computer monitor at 55 cm from the participant.

Every stimulus display included four isoluminant color square frames (2 red and 2 green; Kovesi, 2015) with 1 degree width × 1 degree height and a frame width of 0.1 degrees. The four frames were each located in distinct quadrants within the screen, measuring 5 degrees diagonally from the centrally located fixation point. A single square frame containing a small gap (see Titration Procedure) positioned either on the left or right vertical edge served as the target. The remaining three fully closed square frames served as distractors. The location and color of all stimuli were randomly assigned without replacement, such that two frames were always depicted in red and two in green. The pre-cue was a colored arrow corresponding to one of the two colors with dimensions 0.71 degrees in length and 0.15 degrees in width, pointed toward either the left or right hemifield.

#### Procedure

##### Main experiment

Participants performed the experiment in a dark room while seated comfortably 60 cm from the computer monitor. At the beginning of each trial, a black fixation cross appeared in the center of the screen with dimensions measuring 1 degree length and 0.1 degrees width. Next, four black square frames appeared to demarcate the size and position of each item in the upcoming search display. Following a 1000 ms delay, the black fixation cross was replaced by a 300 ms pre-cue (a colored arrow pointing left or right). The pre-cue accurately predicted the correct hemifield containing the upcoming target frame (space-valid, feature-invalid, or SvFi cue; 10% of all trials), the color of the upcoming target frame (space-invalid, feature-valid, or SiFv cue; 10%), both (space-valid, feature-valid, or SvFv cue; 70%), or neither (space-invalid, feature-invalid, or SiFi cue; 10%; see the Table.

**Table.** Percentage of trials with each cue type in each experiment.

	SvFv	SvFi	SiFv	SiFi	Sv total	Fv total
**Experiment 1**	70%	10%	10%	10%	80%	80%
**Experiment 2a**	10%	40%	40%	10%	50%	50%
**Experiment 2b**	40%	10%	40%	10%	50%	80%

After a variable amount of time (500, 1000, or 1500 ms) from the pre-cue presentation, all black square frames were temporarily changed to three distractors and one target colored either red or green (see Materials and stimuli) for 200 ms. Following a 100 ms blank period, the initial four black square frames and fixation cross reappeared on the screen to act as a backward mask for 1000 ms. Participants were instructed to maintain fixation at center throughout each trial, and their compliance was monitored with an eye tracker (see Eye tracking).

**Figure 1.**
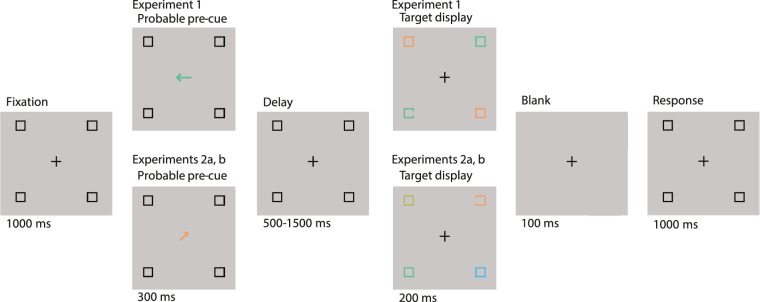
Illustration of a single trial sequence in Experiment 1 (*top*) and Experiment 2 (*bottom*). At the beginning of each trial, a 1000 ms fixation screen was presented and then replaced by a 300 ms central pre-cue providing both color and spatial information about the upcoming target (a square with an opening on either the left or the right side). After a variable delay (500, 1000, or 1500 ms), the target appeared, and participants were instructed to report the position of the opening on the target. The size of the opening was adjusted dynamically according to the participant's performance.

Participants responded to the location of the small gap on the target square frame (left or right vertical edge) by making a button-press (left or right arrow key on a QWERTY keyboard) within a 1500 ms response window from the start of the mask presentation (see Figure 1). Participants completed seven blocks of 80 trials each, for a total of 560 trials. The initial block served as practice and was later removed from subsequent analyses. Because the task was challenging, participants were offered incremental breaks within and between blocks. Performance feedback was provided on each trial and before each break.

##### Titration procedure

To ensure that the target gap was not overly salient such that accurate identification should require on-going attentional deployment, we titrated the size of the gap to keep performance within a relatively low range of 60% to 70% correct (see Liang & Scolari, 2020). Mean accuracy across all pre-cue types was evaluated every 15 trials throughout the length of the experiment. If performance exceeded this range, the gap size was decreased by 20%, down to a minimum length of 0.05 degrees; if performance fell below this range, the gap increased in size by 20% up to a maximum length of 0.5 degrees. Because a single titration procedure was applied to all conditions, the overall mean is expected to fall within the criterion range without constraining whether and how any one condition deviates from the mean.

##### Ishihara color test

Because this study uses color cues, participants first completed a computerized Ishihara color test (Nakajima, Ichikawa, Nakagawa, Majima, & Watanabe, 1960) to confirm normal color vision. Each subject orally reported without time pressure which number or pattern was inserted in a color dot pattern on the screen. Every participant passed this preliminary screening, and this data was excluded from further analyses.

##### Eye tracking

The eye tracker was first calibrated using 13 reference points in the chinrest free mode, after which each participant's right eye gaze position was recorded at a sampling rate of 500 Hz. Offline, an interest period was introduced from pre-cue onset to target offset, and an interest area was defined as a square of 3.33 degrees × 3.33 degrees centered on fixation.

#### Data analyses

##### Preprocessing

Before evaluating attention effects, we first removed trials in which participants blinked, made saccades, or made fixations outside of the interest area during the interest period. Next, we removed trials in which participants selected neither of the two designated response buttons.

##### Titration outcomes

Due to likely differences in perceptual ability, we titrated the size of the target gap throughout the experiment to equate task difficulty across participants, with a single procedure applied to all trial types. Given this approach, we expected the average gap size to be equivalent between cueing conditions (Liang & Scolari, 2020).

##### Attention effects in accuracy

Given that the task included a two-alternative forced choice (2AFC) design and that the pre-cue conveyed no information about the correct response on a given trial (i.e. the location of the gap on the target frame was orthogonal to the cued information), we used a sensitivity index (*d'*) to evaluate accuracy-based attention effects after preprocessing. RT analyses were additionally conducted on correct trials only, calculated from the onset of the response window (see Supplementary Materials).

##### Attention effects in decisional processes

To investigate attention effects within latent decision-making components, we used a robust EZ-diffusion model (Wagenmakers et al., 2007; Wagenmakers et al., 2008; Liang & Scolari, 2020). This is a modification of the Ratcliff diffusion model (Ratcliff, 1978; Ratcliff & McKoon, 2008), which reduces model outputs from seven to three. Although there are several modified diffusion models available that have shown to accurately estimate cognitive processes in binary decision tasks, the EZ model is ideal for the current study given that it works with closed-form equations using few degrees of freedom.

The EZ model takes response accuracy (*P_c_*), mean correct RT (*MRT*), and variance of correct RT (*VRT*) as inputs to produce three estimates: (1) drift rate (*v*), or the speed with which perceptual evidence for one of two response options is accumulated; (2) boundary separation (*a*), or the conservativeness of a response criterion; and (3) non-decision time (*T_er_*), or the proportion of time spent outside of evidence accumulation (Wagenmakers et al., 2007). Additionally, the robust extension to the original EZ model removes contaminated data resulting from attentional lapses (Wagenmakers et al., 2008).

We submitted the data to repeated-measures ANOVAs, as appropriate. Because conclusions regarding independence between SBA and FBA mechanisms may only be supported via a nonsignificant interaction, the results are accompanied by a Bayes factor analysis. To determine the relative strength of evidence for all models that contain the fixed effect of the attention mechanism compared to all those that do not (Clyde, Ghosh, & Littman, 2011), we used an inclusion Bayes factor using the *bayestestR* R package (Makowski, Ben-Shachar, & Lüdecke, 2019). For pairwise comparisons, we used the *BayesFactor* R package (Morey, Rouder, Jamil, & Morey, 2015) to calculate a default Bayes factor with a wide Cauchy distribution (scale of prior distribution = 0.707).

### Results and discussion

#### Preprocessing

To accurately evaluate attention effects, we first removed trials in which participants blinked, made saccades, or fixated outside of the fixation area during the interest period (15.49% of trials on average across subjects). We then removed trials in which neither of the two response buttons was selected (0.75% of the remaining trials). On average, 85.03% of trials across subjects were included for the following analyses.

#### Titration outcomes

**Figure 2.**
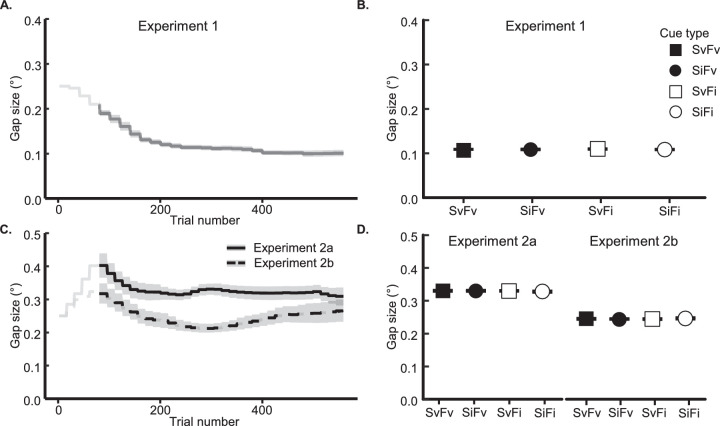
Target gap size (in visual degrees) from Experiment 1 (**A,** **B**) and Experiment 2 (**C,** **D**). The changes in gap size are plotted on a trial-by-trial basis (before preprocessing; **A**: Experiment 1; **C**: Experiment 2). The average gap size for each pre-cue type (SvFv: space-valid, face-valid; SvFi, space-valid, feature-invalid; SiFv, space-invalid, feature-valid; and SiFi, space-invalid, feature-invalid) is plotted (**B**: Experiment 1; **D**: Experiment 2), excluding the practice block, with ±1 within-participant SEM bars.

On average, the gap size gradually decreased to approximately 0.12 degrees by the 220th trial and remained stable for the rest of the task (see Figure 2A-B). A 1-way ANOVA on gap size confirmed that it was well matched across each of the four cue types, *F*(3,90) = 1.10, *p* = 0.35, $\eta_{G}^{2}$ < 0.001, BF_10_ = 0.15. Thus, any attention effects reported below are not driven by stimulus display differences across conditions.

#### Attention effects in accuracy

Attention impacts visual processing by (1) enhancing the signal of relevant information and/or (2) suppressing the signal of cluttered irrelevant information, where present (Dosher & Lu, 2000). Because we used a sparse display, where the distractors were widely separated from the target, we largely attribute attention effects within sensitivity as evidence of signal enhancement.

**Figure 3.**
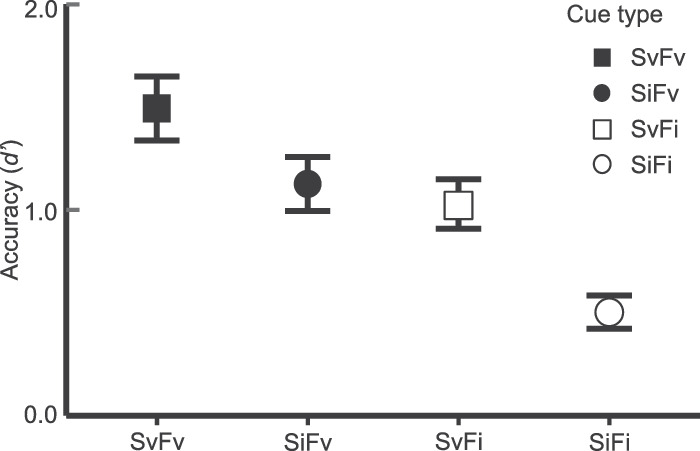
Results from Experiment 1. Mean response accuracy (*d* ′) is plotted with ±1 within-participant SEM error bars for each cue type. Square and circle markers indicate valid and invalid spatial cue components, respectively; filled and open markers indicate valid and invalid feature cue components, respectively.

The average *d'* for each pre-cue type is depicted in Figure 3A. We observed significant attention effects for both cued dimensions (spatial cue: *F*(1, 30) = 16.57, *p* < 0.001, $\eta_{G}^{2}$ = 0.091, BF_10_ = 81.08; feature cue: *F*(1, 30) = 15.34, *p* < 0.001*,* $\eta_{G}^{2}$ = 0.13, BF_10_ > 100). Sensitivity was higher on valid spatial cue trials (M = 1.26) than on invalid spatial cue trials (M = 0.81). The same pattern occurred across feature validity conditions (valid: M = 1.31; invalid: M = 0.77). Importantly, there was no interaction between cue types, *F*(1, 30) = 0.41, *p* = 0.53, $\eta_{G}^{2}$ = 0.003, BF_10_ = 0.31, suggesting that the location and color cues independently influenced sensitivity. This is also supported when we examine accuracy on the SvFv trials (*d* ′ = 1.49), which does not exceed the sum of the SiFv (*d* ′ = 1.13) and SvFi (*d* ′ = 1.03) trials, as you might expect in a super-additive relationship.

#### Attention effects in decisional processes

When each of the feature and spatial pre-cues provided unique information about the upcoming target, both were deployed simultaneously and independently to facilitate accurate identification. Notably, perceptual decision making includes multiple latent elements, such as signal strength, response caution, preprocessing, and processing speed that may be uniquely influenced by attention (Liang & Scolari, 2020; Ratcliffe, 1978; Wagenmakers et al., 2007). Thus, to further investigate how the validity of the two pre-cues modulated these distinct components, we submitted the data to a simple diffusion model.

##### Drift rate (v)

**Figure 4.**
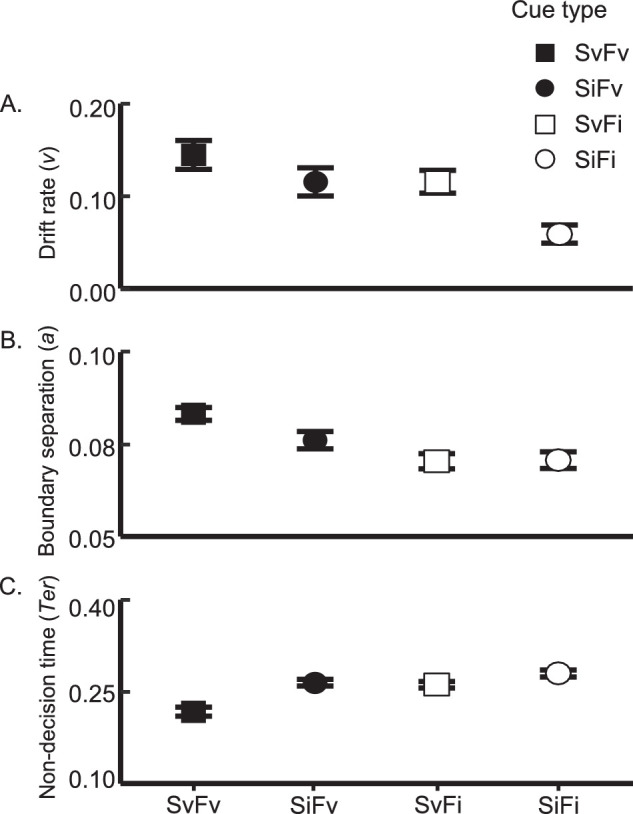
Results from the robust EZ-diffusion model (**A**: drift rate; **B**: boundary separation; and **C**: non-decision time) in Experiment 1 are plotted with ±1 within-participant SEM error bars, as per cue valid and invalid.

Drift rate reflects the speed with which evidence is accumulated from an attended object in favor of one response alternative or the other. We thus consider this measure to be reflective of signal enhancement, much like sensitivity. Figure 4A depicts the average drift rates of each cue type. As we would expect, drift rate followed an identical pattern to that of sensitivity. Both FBA and SBA modulated drift rate (feature: *F*(1, 30) = 8.11, *p* = 0.008, $\eta_{G}^{2}$ = 0.076, BF_10_ = 25.7; spatial: *F*(1, 30) = 13.15, *p* = 0.001, $\eta_{G}^{2}$ = 0.076, BF_10_ = 25.26). On average, perceptual information was accumulated at a faster rate following a valid feature cue (M = 0.13) compared to an invalid feature cue (M = 0.087). Similarly, the accumulation rate following a valid spatial cue (M = 0.13) was greater than that following an invalid cue (M = 0.087). Importantly, the two cue types did not significantly interact, *F*(1, 30) = 1.04, *p* = 0.32, $\eta_{G}^{2}$ = 0.008, BF_10_ = 0.42. Again, this result suggests that color and location information were utilized independently to gather perceptual evidence in favor of one response alternative over another.

##### Boundary separation (a)

Boundary separation indicates how much evidence was accumulated before response initiation. The feature cue significantly modulated boundary separation, *F*(1, 30) = 14.07, *p* < 0.001, $\eta_{G}^{2}$ = 0.11, BF_10_ > 100: valid feature cues (M = 0.08) led to more conservative responses than invalid cues (M = 0.071). The validity of the spatial cue trended in the same direction (valid spatial cue: M = 0.077, invalid spatial cue: M = 0.073), although the effect did not reach significance, *F*(1, 30) = 2.43, *p* = 0.13, $\eta_{G}^{2}$ = 0.017, BF_10_ = 0.65 (see Figure 4B).

Importantly, and in contrast to drift rate, we observed a significant interaction between feature and spatial cue validity, *F*(1, 30) = 5.24, *p* = 0.029, $\eta_{G}^{2}$ = 0.02, BF_10_ = 0.99. The FBA effect was significant when the paired spatial cue component was valid (SvFv versus SvFi cues), *t*(30) = 5.23, *p* < 0.001, *d* = 0.94, BF_10_ > 100, but not when the spatial cue was invalid (SiFv versus SiFi cues), *t*(30) = 1.62, *p* = 0.12, *d* = 0.29, BF_10_ = 0.62. Similarly, a significant SBA effect was only observed when the feature cue component was valid (SvFv vs. SiFv cues), *t*(30) = 2.67, *p* = 0.012, *d* = 0.48, BF_10_ = 3.80, and not when it was invalid (SvFi versus SiFi cues), *t*(30) = 0.11, *p* = 0.91, *d* = 0.02, BF_10_ = 0.19. Thus, participants accumulated more evidence before generating a response following a wholly valid cue compared to other cueing conditions.

##### Non-decision time (T_er_)

Non-decision time reflects processing time that is not directly related to evidence accumulation (e.g. perceptual preparation and motor response). Where all trials are equally likely to end with a left or right arrow button response regardless of cue type, we assume motor response does not meaningfully differ between conditions. Thus, any significant results in non-decision time should reflect perceptual preparation before evidence accumulation onsets, including attentional shifts in response to the stimulus display. Here, both cue types modulated non-decision time (feature cue: *F*(1, 30) = 13.80, *p* < 0.001, $\eta_{G}^{2}$ = 0.056, BF_10_ > 100 ; space cue: *F*(1, 30) = 53.71, *p* < 0.001, $\eta_{G}^{2}$ = 0.069, BF_10_ > 100), such that non-decision time was shorter when each cue was valid (feature: M = 0.24; space: M = 0.22) than when it was invalid (feature: M = 0.27; space: M = 0.25; see Figure 4C).

Consistent with our observations in boundary separation, the feature and spatial cues interacted significantly, *F*(1, 30) = 8.18, *p* = 0.008, $\eta_{G}^{2}$ = 0.015, BF_10_ = 2.75. The FBA effect was significant when the spatial cue was valid (SvFv versus SvFi cues), *t*(30) = 4.39, *p* < 0.001, *d* = 0.79, B_10_ > 100, and not when spatial cue was invalid (SiFv versus SiFi cues), *t*(30) = 1.69, *p* = 0.10, *d* = 0.30, B_10_ = 0.68. The SBA effect was significant whether the feature cue was valid (SvFv versus SiFv cues), *t*(30) = 5.94, *p* < 0.001, *d* = 1.07, B_10_ > 100, or invalid (SvFi versus SiFi cues), *t*(30) = 3.40, *p* = 0.0019, *d* = 0.61, B_10_ = 18.43, although notably the effect was larger for the former case. These results fit well with the boundary results reported above: when a relatively small amount of finite trial time is dedicated to perceptual preparation, the more conservative the response can be, given some fixed drift rate. Hence, evidence accumulation onset sooner and reached a higher level when the pre-cue was wholly valid compared to other cueing conditions.

### Comparisons between experiments

The primary difference between this experiment and Experiment 2 of Liang and Scolari (2020) is that here, each dimension of the pre-cue provided unique information to facilitate target identification. We extend on our previous findings by showing that when the task design sufficiently encourages the simultaneous deployment of space- and feature-based selection, participants use both to perceptually resolve the target in an independent manner, consistent with previous studies (e.g. White et al., 2015). At the same time, we also replicate our previous findings that both cue components are utilized in a dependent manner within perceptual decision-making processes outside of signal enhancement.

Interestingly, removing the redundancy of the cue without changing the validity attached to each pre-cue dimension only impacted measures of signal enhancement: where we were unable to detect evidence of FBA within sensitivity and drift rate using redundant and highly reliable cues (Liang & Scolari, 2020), FBA effects now emerged in these measures. We compared the two experiments directly to determine whether these differences were reliable. A set of 2 (experiment) x 2 (feature validity) x 2 (spatial validity) mixed-design ANOVAs^1^ revealed a significant experiment x feature validity interaction in both *d* ′ (*F*(1, 59) = 12.74, *p* < 0.001, $\eta_{G}^{2}$ = 0.05, BF_10_ > 100) and drift rate (*F*(1, 59) = 6.70, *p* = 0.01, $\eta_{G}^{2}$ = 0.03, BF_10_ = 47.27). Not surprisingly, we observed a significant main effect of the spatial cue in both measures (*d* ′: *F*(1, 59) = 27.13, *p* < 0.001, $\eta_{G}^{2}$ = 0.09, BF_10_ > 100; drift rate: *F*(1, 59) = 23.30, *p* < 0.001, $\eta_{G}^{2}$ = 0.08, BF_10_ > 100), but unlike the feature cue, the size of the effect did not differ between experiments (*d* ′: *F*(1, 59) = 0.32, *p* = 0.35, $\eta_{G}^{2}$ = 0.003, BF_10_ = 0.40; drift rate: *F*(1, 59) = 0.57, *p* = 0.45, $\eta_{G}^{2}$ = 0.002, BF_10_ = 0.25). No other interactions reached significance within either measure (all *p* values > 0.18; all BF_10_s < 0.47).

In contrast, both boundary separation and non-decision time showed statistically identical patterns across the two experiments. Within boundary separation, we observed a main effect for each cue type (feature cue: *F*(1, 59) = 26.76, *p* < 0.001, $\eta_{G}^{2}$ = 0.09, BF_10_ > 100; spatial cue: *F*(1, 59) = 8.26, *p* = 0.006, $\eta_{G}^{2}$ = 0.03, BF_10_ = 6.33) and an interaction between cues, *F*(1, 59) = 13.07, *p* < 0.001, $\eta_{G}^{2}$ = 0.03, BF_10_ = 19.16, consistent with both experimental reports. The same was true for non-decision time (feature cue: *F*(1, 59) = 22.54, *p* < 0.001, $\eta_{G}^{2}$ = 0.02, BF_10_ > 100; spatial cue: *F*(1, 59) = 68.08, *p* < 0.001, $\eta_{G}^{2}$ = 0.05, BF_10_ > 100; feature x spatial cue: *F*(1, 59) = 12.49, *p* < 0.001, $\eta_{G}^{2}$ = 0.01, BF_10_ = 33.84). However, we did not observe significant differences between experiments for either component or their interaction with cue type (all *p* values > 0.24; all BF_10_s < 0.45).

This provides further evidence of the separability of signal enhancement and other latent perceptual decision-making processes, even in the same visual search task. Removing the redundancy of the cue components while maintaining high SvFv cue frequency impacted participants’ use of feature information to perceptually resolve the target but did little to change evidence accumulation onset time or the amount of evidence required to initiate a response. Given that the probability of each cueing object was held constant across the two experiments, we consider the possibility that the likelihood of a given cue, and not its unique contributions, modulates perceptual decision making outside of signal enhancement. Experiments 2a and 2b test this possibility by manipulating the probabilities attached to each cueing object.

## Experiment 2a

Experiment 1 showed that within our paradigm, we can observe independent SBA and FBA effects in signal enhancement measures as well as super-additive effects in decision-making components when each dimension of the pre-cue uniquely contributes to target search. Returning to our redundant cueing approach, we next examined whether and how SBA and FBA interact when each pre-cue dimension predicts the upcoming target with equally moderate reliability. Because White and colleagues (2015) detected FBA effects using moderately reliable cues, we expected this decrease in validity would allow us to again observe independent SBA and FBA effects while also investigating how shifts in cue probability might influence different aspects of perceptual decision making.

### Methods

#### Participants

A power analysis (Mayr, Erdfelder, Buchner, & Faul, 2007) assuming a medium effect size ($\eta_{p}^{2}$ = 0.09) and moderate within-subject correlation (rho = 0.6) produced a target sample size of 16 participants to detect effects with 80% power and 5% false discovery rate. Ultimately, 20 undergraduate students (6 men and 14 women) were recruited via the Texas Tech University Research Participation System (SONA) and received course credit for their full participation. All participants gave written informed consent in accordance with both the requirements of the Institutional Review Board and the Declaration of Helsinki. Each participant had normal or corrected-to-normal vision, and normal color vision as determined by an Ishihara Color Test (Nakajima et al., 1960).

#### Materials and stimuli

The materials and stimuli closely matched those reported in Experiment 1. However, we increased the number of isoluminant colors from two to four (red, yellow, blue, and green), and the pre-cue arrow pointed to one of four quadrants rather than hemifields. These adjustments matched the stimulus parameters described in Liang and Scolari (2020).

#### Procedure

##### Main experiment

The procedure for the main experiment closely matched that of Experiment 1, with some notable differences. Here, the spatial and feature pre-cues were each solely valid 40% of the time (SvFi and SiFv trials), both dimensions of the cue were valid 10% of the time (SvFv trials), and both dimensions were invalid 10% of the time (SiFi trials). This means the spatial and the feature components were each valid 50% of the time. Given that the sparse search display included four items, note that a cue with 50% validity provides reliable information about the relevant target-containing item. See the Table for the cue breakdown and Figure 1 for an illustration of a single trial.

##### Titration procedure

See Experiment 1 Methods for a description of the titration procedure used here.

##### Eye tracking

See Experiment 1 Methods for a description of the eye-tracking procedures used here.

#### Data analyses

See Experiment 1 Data Analyses for a description of the analyses used here.

### Results and discussion

#### Preprocessing

To accurately evaluate attention effects in the current study, we first removed trials in which participants blinked, made saccades, or made fixations outside of the interest area during the interest period (11.20% of trials on average). Next, we removed trials in which the participant failed to select either of the two designated response buttons (1.37% of the remaining trials). On average, 78.40% of trials from each participant survived preprocessing.

#### Titration outcomes

On average, gap size first approached the maximum value during the initial block, indicating that participants needed practice to become familiar with the task. After this period, the gap size decreased and eventually remained stable at around 0.31 degrees (see Figure 2C). Importantly, we confirmed that the gap size did not differ significantly across the four different pre-cue types, *F*(3, 57) = 0.61, *p* = 0.61, $\eta_{G}^{2}$ < 0.001, BF_10_ = 0.13 (see Figure 2D).

#### Attention effects in accuracy

**Figure 5.**
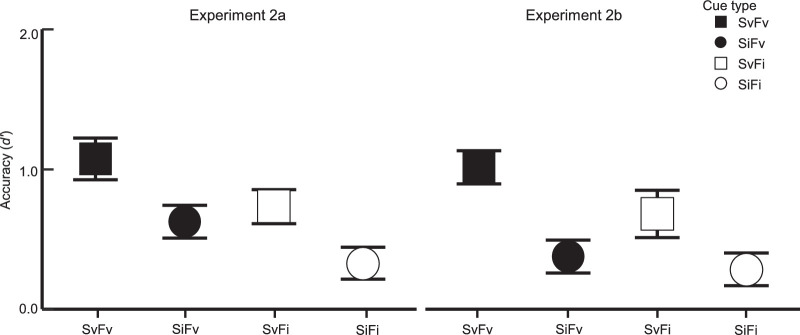
Results from Experiments 2a and 2b. Mean response accuracy is plotted with ±1 within-participant SEM error bars.

Mean *d* ′ for each trial type is depicted in Figure 5A. The validity of the spatial cue significantly modulated *d* ′, *F*(1, 19) = 7.13, *p* = 0.015, $\eta_{G}^{2}$ = 0.099, BF_10_ = 29.34. Accuracy was higher when the spatial cue was valid (M = 0.90) than when it was invalid (M = 0.48). Consistent with Experiment 1, accuracy depended on the validity of the feature cue as well, *F*(1, 19) = 6.32, *p* = 0.021, $\eta_{G}^{2}$ = 0.058, BF_10_ = 3.81. Accuracy was higher when the feature cue was valid (M = 0.85) than when it was invalid (M = 0.53). We again did not observe a significant interaction between cue types, *F*(1, 19) = 0.08, *p* = 0.78, $\eta_{G}^{2}$ < 0.001, BF_10_ = 0.32. This straight-forward pattern of results indicates that participants again utilized both cues to identify the target in an independent, additive manner (White et al., 2015). This is further supported when we examine accuracy on the SvFv trials (*d'* = 1.07), which is less than the sum of the SiFv (*d'* = 0.63) and SvFi (*d'* = 0.73) trials.

#### Attention effects in decisional processes

The behavioral performance results described above suggest that participants utilized both dimensions of the pre-cue independently to facilitate target identification, consistent with previous reports (Ásgeirsson, Kristjánsson, & Bundesen, 2014; White et al., 2015). Given the sparse nature of our stimulus display, we broadly consider accuracy to be an indirect measure of the signal enhancing effects of attention. We next set out to estimate attention effects on underlying factors that uniquely contribute to perceptual decision making using the robust EZ-diffusion model.

##### Drift rate (*v*)

**Figure 6.**
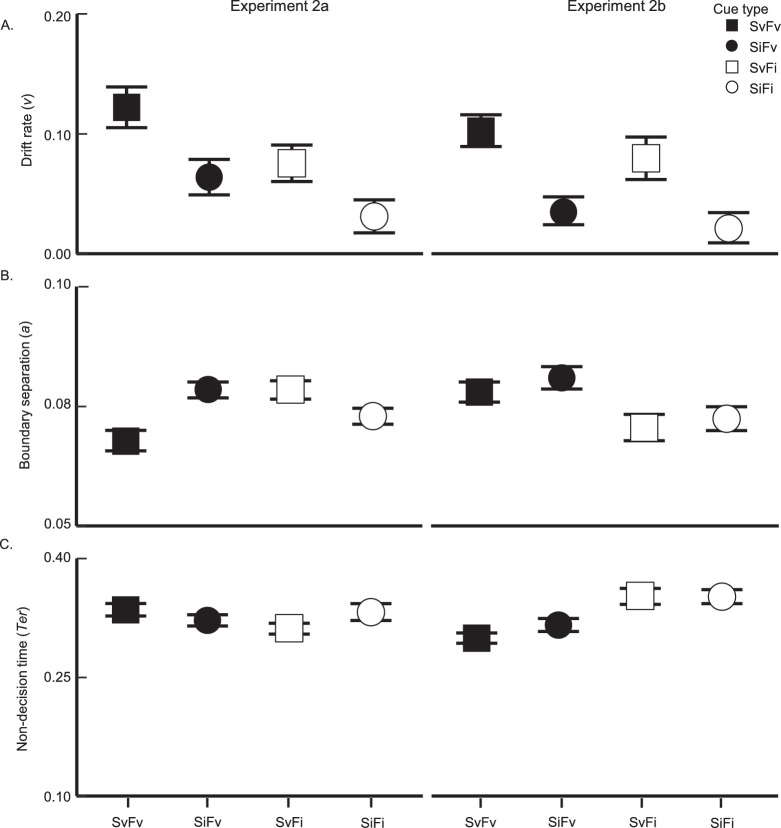
Results from the robust EZ-diffusion model (**A**: drift rate; **B**: boundary separation; and **C**: non-decision time) in Experiment 2 are plotted with ±1 within-participant SEM error bars.

Figure 6A shows the average drift rate for each cue type. Echoing the accuracy results above, we found a significant main effect of both the spatial cue, *F*(1, 19) = 7.16, *p* = 0.015, $\eta_{G}^{2}$ = 0.10, B_10_ = 29.71, and the feature cue, *F*(1, 19) = 6.41, *p* = 0.02, $\eta_{G}^{2}$ = 0.065, BF_10_ = 4.64, but no evidence of an interaction, *F*(1, 19) = 0.58, *p* = 0.46, $\eta_{G}^{2}$ < 0.002, BF_10_ = 0.34. Moreover, the drift rate for SvFv trials (M = 0.12) was roughly equivalent to the sum of the SvFi (M = 0.08) and SiFv trials (M = 0.06), consistent with an additive relationship between the two cue types within evidence accumulation.

##### Boundary separation (*a*)

Interestingly, the most conservative responses occurred on trials in which only one pre-cue dimension was valid (SvFi: M = 0.078; SiFv: M = 0.078) compared to wholly valid or invalid trials (SvFv: M = 0.068; SiFi: M = 0.073). This resulted in no main effect of either pre-cue (feature cue: *F*(1, 19) = 2.29, *p* = 0.15, $\eta_{G}^{2}$ = 0.012, BF_10_ = 0.44; spatial cue: *F*(1, 19) = 1.42, *p* = 0.25, $\eta_{G}^{2}$ = 0.012, BF_10_ = 0.43), but a significant cross-over interaction between the two pre-cues, *F*(1, 19) = 21.92, *p* < 0.001, $\eta_{G}^{2}$ = 0.11, BF_10_ > 100 (see Figure 6B). The FBA effect was only significant when the paired spatial cue component was invalid (SiFv versus SiFi cues), *t*(19) = 2.79, *p* = 0.012, *d* = 0.63, BF_10_ = 4.52. When the paired spatial cue was valid, we observed a significant reversal of the FBA effect (SvFv versus SvFi cues), *t*(19) = −3.81, *p* = 0.0012, *d* = 0.85, BF_10_ = 31.88. A similar pattern occurred for the SBA effect: we observed a marginally significant effect when the paired feature cue was invalid (SvFi versus SiFi cues), *t*(19) = 2.08, *p* = 0.051, *d* = 0.47, BF_10_ = 1.37, and a significant reversal when the feature cue was valid (SvFv versus SiFv cues), *t*(19) = −3.72, *p* = 0.0015, *d* = 0.83, BF_10_ = 26.21.

##### Non-decision time (*T_er_*)

A similar crossover interaction was detected within non-decision time, *F*(1, 19) = 5.13, *p* = 0.035, $\eta_{G}^{2}$ = 0.011, BF_10_ = 1.71 (no main effects; feature cue: *F*(1, 19) = 0.89, *p* = 0.36, $\eta_{G}^{2}$ = 0.002, BF_10_ = 0.31; spatial cue: *F*(1, 19) = 0.15, *p* = 0.70, $\eta_{G}^{2}$ < 0.001, BF_10_ = 0.25). We again observed a significant reversal of the FBA effect when the paired spatial cue was valid (SvFv versus SvFi), *t*(19) = 2.89, *p* = 0.0095, *d* = 0.65, BF_10_ = 5.37, such that evidence accumulation onset earlier following the SvFi cue (M = 0.31) than the SvFv cue (M = 0.34). No other planned comparisons reached significance (all *p* values > 0.14; all BF_10_s < 0.63).

In line with previous studies (Liang & Scolari, 2020; White et al., 2015), our results show that participants deployed SBA and FBA independently to perceptually resolve the target, as reflected by accuracy and drift rate. Consistent with Experiment 1 and Liang and Scolari (2020), significant interactions also emerged between the feature and spatial cues within both boundary separation and non-decision time. However, the patterns within each process differed from our previous observations. When the SvFv cue was highly probable (70%), it elicited relatively shorter non-decision times and more conservative responses. Here, the SvFi and SiFv cues were relatively more probable (40% each) compared to the others, and these cues elicited more conservative responses and, to a lesser extent, shorter non-decision times. The results lead us to tentatively conclude that these decision-making components depend on the probability of the whole cueing object, in contrast to measures of signal enhancement, which depends on each component part in isolation. This was especially true within boundary separation, whereas the pattern was somewhat consistent but less reliable in non-decision time. Experiment 2b continues to explore this possibility by manipulating cue likelihoods again.

## Experiment 2b

We continued to investigate how changing the likelihood of a whole cueing object may differentially impact attention-related signal enhancement and other decision-making mechanisms. Compared to Experiment 2a, probabilities were redistributed across cue types in the following manner: the probabilities of both the SiFv and SiFi cues remained fixed at 40% and 10% of all trials, respectively. Additionally, the probability attached to the SvFv cue increased to 40% and the SvFi cue decreased to 10%. Thus, cueing objects containing a valid feature occurred more frequently than those containing an invalid feature regardless of whether they were paired with a valid or invalid spatial cue. In contrast, the cueing objects containing a spatially valid component were no more or less frequent than their spatially invalid counterparts.

Because both cue components themselves are reliable to the same extent they were in the previous experiments (i.e. the feature cue is valid 80% of the time and the spatial component is valid 50% of the time when summed across whole cueing objects), we expect to again observe significant, independent attention effects within signal enhancement. If boundary separation and non-decision time depend on the likelihood of the whole cueing object rather than the reliabilities of the component parts, we expect to see evidence of FBA effects in these measures, but no SBA effect and no interaction, in a departure from both signal enhancement and the previous experiment.

### Methods

#### Participants

Twenty undergraduate students (5 men and 15 women) with normal or corrected-to-normal vision were recruited via the Texas Tech University Research Participation System (SONA) to match the number of participants in Experiment 2a. Each participant gave written informed consent as required by the Institutional Review Board and in compliance with the Declaration of Helsinki and received partial course credit for their participation. All participants passed the Ishihara color test (see Experiment 1 Methods) for normal color vision screening.

#### Materials and stimuli

The materials and stimuli were identical to those reported in Experiment 2a.

#### Procedure

##### Main experiment

The procedure in Experiment 2b matched those reported in Experiment 2a (see Figure 1), except for the following changes to the cueing validity. For 40% of the trials, the color of the pre-cue matched the color of the upcoming target (SiFv); for 10% of the trials, the direction of the pre-cue accurately predicted the location of the upcoming target (SvFi); for 40% of the trials, the pre-cue accurately predicted both the spatial location and the color of the upcoming target (SvFv); for remaining 10% of the trials, neither the direction or the color of the pre-cue predicted the following target (SiFi). Thus, the feature component of the cue was more reliable (80% valid in total) than the spatial component (50% valid in total; see the Table).

##### Titration procedure

See Experiment 1 Methods for a detailed description.

##### Eye tracking

See Experiment 1 Methods for detailed eye tracker settings and procedures.

#### Data analyses

See Experiment 1 Methods for a description of the data analyses and procedures.

### Results and discussion

#### Preprocessing

We applied the same preprocessing procedures as in Experiments 1 and 2a. We removed 10.70% of trials across participants due to blinks, saccades, and fixations outside of the interest area during the interest period. Of those remaining, another 0.98% of trials were removed due to undesignated response button selection. Thus, on average, 78.60% of trials across participants were survived for the following analyses.

#### Titration outcomes

Gap size reached the maximum level by the end of the practice block (the first 80 trials), and then dropped to 0.22 degrees by the 300th trial (see Figure 2C). However, likely due to fatigue, the gap size increased again to 0.28 degrees by the end of the experiment. Importantly though, across the length of the experiment, gap size was statistically matched across all cueing objects (SvFi, SiFv, SvFv, and SiFi), *F*(3, 57) = 0.45, *p* = 0.72, $\eta_{G}^{2}$ < 0.001, BF_10_ = 0.11 (see Figure 2D).

#### Attention effects in accuracy

Figure 5A depicts the average *d'* for each trial type. As expected, we observed main effects for both cue types (spatial cue: *F*(1, 19) = 7.90, *p* = 0.011, $\eta_{G}^{2}$ = 0.13, BF_10_ = 133.96; feature cue: *F*(1, 19) = 5.34, *p* = 0.032, $\eta_{G}^{2}$ = 0.025, BF_10_ = 0.72), and no evidence of an interaction, *F*(1, 19) = 1.41, *p* = 0.25, $\eta_{G}^{2}$ = 0.008, BF_10_ = 0.32. The average *d'* for SvFv trials (M = 1.01) was roughly equivalent to the sum of SvFi (M = 0.68) and SiFv trials (M = 0.38), suggesting an additive effect of using both pre-cues for target identification.

#### Attention effects in decisional processes

##### Drift rate (*v*)

Drift rate was greatest for SvFv trials (M = 0.11), followed by SvFi trials (M = 0.08), SiFv trials (M = 0.04), and finally SiFi trials (M = 0.02; see Figure 6A). The validity of the spatial pre-cue modulated the rate at which participants accumulated perceptual evidence, *F*(1,19) = 10.50, *p* = 0.004, $\eta_{G}^{2}$ = 0.18, BF_10_ > 100. Surprisingly, the effect of the feature cue was only marginally significant, without clear evidence to support either the alternative or null hypotheses, *F*(1, 19) = 3.15, *p* = 0.092, $\eta_{G}^{2}$ = 0.019, BF_10_ = 0.55. This contrasts with the significant FBA effect reported for accuracy. However, the effect became significant when fast responses were removed (made 250 ms after the onset of the response window; Wagenmakers et al., 2008), *F*(1,19) = 5.02, *p* = 0.037, $\eta_{G}^{2}$ = 0.030, BF_10_ = 0.86. Once again, there was no interaction between the two pre-cues, *F*(1, 19) = 0.18, *p* = 0.67, $\eta_{G}^{2}$ = 0.001, BF_10_ = 0.47, indicating SBA and FBA functioned independently during evidence accumulation.

##### Boundary separation (*a*)

On average, responses were more conservative for SiFv trials (M = 0.081), followed by SvFv trials (M = 0.078), SiFi trials (M = 0.072), and then SvFi trials (M = 0.071). We only observed a main effect of the feature cue, *F*(1, 19) = 6.99, *p* = 0.016, $\eta_{G}^{2}$ = 0.075, BF_10_ = 21.62, such that a valid feature cue increased the amount of perceptual evidence required before making a response whether it was paired with a valid spatial cue (SvFv versus SvFi cues: *t*(19) = 2.04, *p* = 0.056, *d* = 0.46, BF_10_ = 1.28) or an invalid one (SiFv versus SiFi cues: *t*(19) = 2.41, *p* = 0.026, *d* = 0.54, BF_10_ = 2.31). We did not observe a significant SBA effect, *F*(1, 19) = 1.19, *p* = 0.29, $\eta_{G}^{2}$ = 0.007, BF_10_ = 0.34, nor an interaction between the two cue types, *F*(1, 19) = 0.08, *p* = 0.78, $\eta_{G}^{2}$ < 0.001, BF_10_ = 0.29 (see Figure 6B).

##### Non-decision time (*T_er_*)

Figure 6C shows the average non-decision time for each pre-cue. Non-decision time was shortest for SvFv trials (M = 0.30), followed by SiFv trials (M = 0.32) and SvFi trials (M = 0.35). We again observed only a significant FBA effect, *F*(1, 19) = 19.64, *p* < 0.001, $\eta_{G}^{2}$ = 0.048, BF_10_ > 100, where non-decision time was significantly shorter following a valid feature cue compared to an invalid feature cue regardless of spatial cue validity (SvFv versus SvFi cues: *t*(19) = 4.30, *p* < 0.001, *d* = 0.96, BF_10_ = 83.76; SiFv versus SiFi cues: *t*(19) = 2.86, *p* = 0.01, *d* = 0.64, BF_10_ = 5.11). A main effect of the spatial pre-cue (*F*(1, 19) = 1.02, *p* = 0.32, $\eta_{G}^{2}$ = 0.002, BF_10_ = 0.32), and an interaction (*F*(1, 19) = 1.32, *p* = 0.27, $\eta_{G}^{2}$ = 0.002, BF_10_ = 0.47) were both absent.

### Comparisons between experiments

In Experiment 2a, both spatial and feature pre-cue components predicted the upcoming target with equivalent reliability, and we observed that SBA and FBA uniquely contributed to the perceptual enhancement of the target. In contrast, both selection mechanisms contributed to the level of response caution in a dependent manner: a valid cue in either dimension elicited an elevated boundary separation only when paired with an invalid cue. At first glance, this seemingly runs counter to what we have observed previously in similar experiments (Experiment 1; Liang & Scolari, 2020), where boundary separation was greatest for trials with a wholly valid cue. However, we note that in all experiments, the most likely cue(s) among the set consistently elicited more conservative responses, indicating that response caution depends on the whole cueing object rather than its component parts. When we altered the cue probabilities again in Experiment 2b, the pattern within boundary separation predictably changed consistent with this conclusion, and fully emerged for non-decision time as well. To determine whether the patterns observed in Experiments 2a and 2b are meaningfully different, we conducted a 3-way ANOVA (experiment × feature cue × spatial cue) on each of the estimates. This analysis allows us to draw firmer conclusions about the role of cue likelihood on specific aspects of perceptual decision making.

Within accuracy, as measured by sensitivity (*d'*), we observed main effects for both pre-cue components (feature cue: *F*(1, 38) = 11.51, *p* = 0.002, $\eta_{G}^{2}$ = 0.04, BF_10_ = 8.88; spatial cue: *F*(1, 38) = 15.0, *p* < 0.001, $\eta_{G}^{2}$ = 0.12, BF_10_ > 100), and statistically similar patterns across the two experiments (experiment × feature cue, *F*(1, 38) = 0.47, *p* = 0.50, $\eta_{G}^{2}$ = 0.002, BF_10_ = 0.26; experiment × spatial cue, *F*(1, 38) = 0.14, *p* = 0.71, $\eta_{G}^{2}$ = 0.001, BF_10_ = 0.26; experiment × feature cue × spatial cue, *F*(1, 38) = 0.59, *p* = 0.45, $\eta_{G}^{2}$ = 0.001, BF_10_ = 0.41). Interestingly, this suggests that changes to the reliability of the feature cue had no impact on how it influenced signal enhancement (see Figure 5A). We return to this point in the General Discussion.

Like we saw with sensitivity, we observed main effects for both cue types within drift rate (feature cue: *F*(1, 38) = 9.55, *p* = 0.004, $\eta_{G}^{2}$ = 0.04, BF_10_ = 7.46; spatial cue: *F*(1, 38) = 17.51, *p* < 0.001, $\eta_{G}^{2}$ = 0.14, BF_10_ > 100). Furthermore, we did not observe any significant differences between experiments, despite the nonsignificant FBA effect in Experiment 2b (experiment × feature cue, *F*(1, 38) = 1.25, *p* = 0.27, $\eta_{G}^{2}$ = 0.005, BF_10_ = 0.35; experiment × spatial cue, *F*(1, 38) = 0.17, *p* = 0.69, $\eta_{G}^{2}$ = 0.002, BF_10_ = 0.36; experiment × feature cue × spatial cue, *F*(1, 38) = 0.03, *p* = 0.86, $\eta_{G}^{2}$ < 0.001, BF_10_ = 0.32). This again suggests that the changes we made to cue validity did not detectably affect signal enhancing effects of space- or feature-based attention (see Figure 6A), and therefore the unreliable FBA effect in Experiment 2b should be interpreted with caution.

In contrast, we did observe a significant three-way interaction within boundary separation, *F*(1, 38) = 8.26, *p* = 0.007, $\eta_{G}^{2}$ = 0.021, BF_10_ = 3.7, indicating the significant interaction between SBA and FBA observed in Experiment 2a reliably differed from the absent interaction in Experiment 2b. Recall that in the latter, boundary separation largely depended on the feature cue. An experiment × feature cue interaction indicated that the significant FBA effect in Experiment 2b reliably differed from the absent main effect in Experiment 2A, *F*(1, 38) = 9.29, *p* = 0.004, $\eta_{G}^{2}$ = 0.04, BF_10_ = 28.44.

To determine which cueing object(s) were the driving force behind these interaction effects, we compared attention effects within boundary separation for each paired cue condition. Recall that in Experiment 2b, we observed a significant FBA effect on space-valid trials (SvFv-SvFi), where SvFv cues were present on 40% of trials and SvFi cues were present on 10% of all trials. Where the probabilities were reversed (Experiment 2a), a reliable reversal was detected. These patterns differed significantly, *t*(35.52) = 3.93, *p* < 0.001, *d* = 1.24, BF_10_ = 38.77. Similarly, the SBA effect reversal detected on feature-valid trials (SvFv-SiFv) in Experiment 2a significantly differed from the absent effect in Experiment 2b, *t*(36.83) = 2.05, *p* = 0.047, *d* = 0.65, BF_10_ = 0.82. In contrast, both the SiFv and SiFi cues were assigned fixed probabilities across experiments (40% and 10%, respectively), and, as expected, the difference scores in boundary separation were statistically matched, *t*(29.65) = 0.75, *p* = 0.46, *d* = 0.24, BF_10_ = 0.32.

A similar pattern occurred within non-decision time: we again observed a significant three-way interaction, *F*(1, 38) = 5.88, *p* = 0.02, $\eta_{G}^{2}$ = 0.005, B_10_ = 2.08, and a significant experiment × feature cue two-way interaction, *F*(1, 38) = 17.24, *p* < 0.001, $\eta_{G}^{2}$ = 0.019, B_10_ > 100, indicating that the FBA effect was significantly larger in Experiment 2b. Echoing boundary separation, the size of the FBA effect on space-valid trials differed significantly between experiments (SvFv-SvFi), *t*(33.30) = 5.18, *p* < 0.001, *d* = 1.64, BF_10_ > 100, as did the SBA effect on feature-valid trials (SvFv-SiFv), *t*(35.22) = 2.22, *p* = 0.033, *d* = 0.70, BF_10_ = 1.12, whereas the FBA effect on space-invalid trials was statistically matched (SiFv-SiFi), *t*(37.97) = 1.46, *p* = 0.15, *d* = 0.46, BF_10_ = 0.85. These results are all in line with the relative probabilities assigned to each cueing object between experiments.

## General discussion

In a recent study, we argued that previously observed dependencies between SBA and FBA may be restricted to components of perceptual decision making related to, but outside of, signal enhancement. These may include: (1) attentional shifts between internal representations that occur before evidence accumulation, and (2) the amount of accumulated evidence preceding response selection (Liang & Scolari, 2020). Notably, though, we were unable to detect an FBA effect within accuracy or drift rate, leading us to use indirect evidence to conclude that attention effects may operate independently within signal enhancement. Here, we rectified this by making a series of singular adjustments to our task to elicit independent FBA and SBA effects in accuracy and drift rate, while still observing dependence in boundary separation and non-decision time.

In Experiment 1, we elicited both attention effects by using an integrated cueing object in which each dimension made unique contributions to the search task. Using just one dimension cut the search space in half but using both in conjunction would reduce it to the single target item on the highly frequent (70%) wholly valid trials. Next, we returned to the redundant cue but reduced the frequency of the wholly valid cue (Experiments 2a and 2b) and increased the relative reliability of the feature dimension (Experiment 2b) so that the likelihood of attending the target item was much greater when using both cue dimensions independently as opposed to just one. All modifications resulted in significant FBA effects within accuracy while also producing an independent SBA effect. Together, these studies provide strong, converging evidence that space- and feature-based attention operate on sensory representations in an independent manner, consistent with other studies (Goddard, Carlson, & Woolgar, 2022; Greenberg et al., 2010; Ni & Maunsell, 2019), and regardless of how probabilities are distributed across integrated cueing objects.

In contrast, we observed dependencies between both selection mechanisms within estimates of boundary separation and non-decision time. In Experiment 1, we observed the shortest non-decision time and greatest response caution when both dimensions of the pre-cue were valid, which occurred on 70% of all trials. These patterns were statistically matched with what we observed previously (Liang & Scolari, 2020, Experiment 2), where the same cueing reliabilities were used. Thus, we successfully replicated our previous findings, again giving more credence to our conclusions that the attention mechanisms interact within specific components of perceptual decision making.

Next, we showed that we could push the interactive patterns around by manipulating the probabilities assigned to each cueing object, despite these same manipulations having no detectable impact on sensory enhancement measures. When the two single-valid cueing objects (SvFi and SiFv) were proportionately most likely, they were followed by abbreviated preparatory processes and more cautious responses compared to other cue types (Experiment 2a), in a departure from what we observed in Experiment 1. This interaction was then eliminated when the distribution of cueing object probabilities was altered again to favor the feature component: in that case, only FBA effects were observed in boundary separation and non-decision time, regardless of the validity of the spatial cue (Experiment 2b). Furthermore, the influence of the spatial cue differed significantly from the previous experiment despite no changes to the total validity of the spatial component itself, but only to its breakdown across whole cueing objects. This result co-occurred with a significant SBA effect in accuracy and drift rate of a fixed size across experiments, indicating that participants were using the spatial cue component to aid target identification to the same degree in both Experiments 2a and 2b.

In sum, we conclude that sensory enhancement depends on the validity of each cued dimension, independently from each other, during perceptual decision-making in a sparse visual search task. However, preparatory processes that occur before evidence accumulation onsets—including attentional shifts between item representations, and the amount of evidence that is acquired before a final decision is executed, depend on both cued dimensions integrated together in a single object. This has critical implications for research on feature- and space-based attention effects, especially by revealing how potentially disparate conclusions from studies relying on distinct measures of accuracy and/or RT can be integrated into a single descriptive model (Liang & Scolari, 2020; White et al., 2015). Here, we demonstrate that these measures can sometimes lead to inconsistent conclusions about whether and how these selection mechanisms interact precisely because they tap into somewhat distinct, albeit related, processes.

Here, we keep to the same general distinction between measures within and outside of sensory enhancement that we established previously (Liang & Scolari, 2020). Specifically, we consider sensitivity and drift rate to both be indicative of signal modulation, where in our sparse display task this should be largely dominated by signal enhancement with little to no distractor suppression. Greater enhancement should result in sharper or more vivid representations, which in turn should (1) elicit steeper accumulation slopes — as relatively more sensory evidence can be gathered per unit time, and (2) facilitate higher perceptual sensitivity. Grouping these measures together as indicative of a common mechanism is further supported by the fact that they have largely shown matching patterns in five different experiments (Liang & Scolari, 2020, Experiments 1 and 2, and the three presented here).

In contrast, non-decision time is defined as pre- and post-decisional processes (van Ravenzwaaij, Dutilh, & Wagenmakers, 2012), squarely outside of sensory enhancement. Because post-decisional processes (e.g. button-presses) are not expected to differ between cueing conditions, we attribute any meaningful cueing differences to initial preparatory activity. This can include any attentional shifts to the target-containing item (or internal representation, as the case may be) that must occur before evidence accumulation in favor of a leftward or rightward gap can begin. Non-decision time and boundary separation consistently showed inverse patterns across most of our experiments, leading us to conclude that shorter non-decision times may generally allow for more conservative responses by quickly giving way to evidence accumulation. We have thus grouped them as indicative of closely related mechanisms, apart from sensory enhancement. It is worth noting, however, that the three perceptual decision-making components, although distinct, are inter-related. We have described here how boundary separation can be inversely dependent on non-decision time; Similarly, it can be positively associated with drift rate (e.g. Pirrone, Dickinson, Gomez, Stafford, & Milne, 2017), where faster evidence accumulation leads to more conservative responses, although that is less evident in our studies. It is possible that boundary separation is more consistent with non-decision time in tasks with time-limited displays that encourage speeded responses, like the ones presented here. Other task designs — perhaps ones without speed pressure — could potentially elicit a stronger relationship with drift rate. In which case, our conclusions about boundary separation may be task dependent.

Our results to date support the conclusion that preparatory decision-making processes and response caution track the likelihood of the whole cueing object. However, because we have always used a single cue containing both spatial and feature information, we cannot rule out the possibility that two distinct spatial and feature cues would produce the same dependent patterns. Future research would benefit from comparing a single two-dimensional cue to two disparate unidimensional cues to determine whether unified objecthood, per se, is a necessary precursor to the cueing effect interactions we observed here. This may be particularly important, given that previous investigations of SBA and FBA interactions have varied between using a single, integrated cue (e.g. Stoppel, Boehler, Sabelhaus, Heinze, Hopf, & Schoenfeld, 2007) and two unique, simultaneously presented cues (e.g. Bengson et al., 2012). Interestingly, Bengson et al. did not find evidence of a dependency between distinct space and feature cues within the P1 and N1 ERP components, each of which occur early in visual processing and are modulated by SBA.

Although the data largely conformed to our primary hypotheses, we note an unexpected finding that deserves closer scrutiny in future research. First, we anticipated that we would observe significant, independent FBA effects within signal enhancement measures when we made the feature dimension of the cue more reliable (Huang et al., 2018; White et al., 2015). We did not anticipate that, once the FBA effect emerged, it would remain stable across both moderate and high cue reliabilities. However, this is what we observed: the FBA effect remained at statistically similar magnitudes whether the informative feature cue was 50% valid or 80% valid. Furthermore, even at 80% valid, the magnitude of the effect was somewhat unreliable and did not exceed that of a 50% valid spatial cue. Combined with our previous work (Liang & Scolari, 2020), this potentially suggests an all-or-nothing deployment of FBA: once FBA emerges, increasing cue validities may not alter its magnitude. This contrasts with several demonstrations in the SBA literature showing that the size of the effect scales with the reliability of a spatial cue (O'Bryan & Scolari, 2021; Riggio & Kirsner, 1997; Vossel et al., 2014), perhaps serving as an additional indicator that SBA and FBA distinctly operate on sensory signals (e.g. Ni & Maunsell, 2019). Notably, there are far fewer investigations of similar scaling effects within the FBA literature. In one such demonstration, Huang and colleagues (2018) showed that a feature cue with 50% validity (uninformative in their two-alternative task) did not produce a significant attention effect within RT, but an 80% valid cue did. In hindsight, this result may be consistent with our own findings, and with the possibility that the scaling effects observed in SBA are not present in FBA.

Notably, this absent scaling in FBA might be specific to a visual search task, where a cued feature is used to guide attention to the location of a target item to facilitate accurate judgments about an orthogonal element (in our paradigm, the position of a small gap; Yeshurun, Montagna, & Carrasco, 2008). The use of a visual search task that inherently relies on item location might also be the reason why the SBA effect is apparently stronger and more robust across manipulations. It would be therefore worthwhile to similarly explore interactions between SBA and FBA in other common attention tasks.

## Conclusion

Our results consistently show that both FBA and SBA are deployed independently to perceptually resolve a target via signal enhancement, indicating unique mechanisms at the level of sensory processing. Within other, higher-order perceptual processes, however, the two attention mechanisms exhibit a dependent relationship. Furthermore, by manipulating the reliability of the pre-cue, we reveal the nature of this dependency: preparatory decision-making processes and response caution track the likelihood of the whole, integrated cueing object rather than its component parts.

## Supplementary Material

Supplement 1

## Footnotes

1 The preprocessing steps differed slightly between the two experiments, and so we applied the same approach described in the current paper to the Liang & Scolari (2020) dataset for these analyses. However, all results and conclusions hold regardless of which set of preprocessing steps are used.
