# Supplementary material for: Space- and feature-based attention operate both independently and interactively within latent components of perceptual decision making
Source: J Vis. 2023 Jan 19;23(1):12. doi: 10.1167/jov.23.1.12 (PMC9872836; doi:10.1167/jov.23.1.12)
Supplement: Supplement 1 [file jovi-23-1-12_s001.pdf]

Supplementary Material

Reaction time (RT) as a single measure includes multiple underlying elements of decision-making such as signal strength, response caution, preprocessing, and processing speed (Ratcliffe, 1978; Wagenmakers et al., 2007; Liang & Scolari, 2020), each of which is separably estimated via the drift diffusion model presented in the main text. We therefore refrain from drawing strong conclusions from the RT analyses but present them here from each experiment for completeness.

Experiment 1

Within RT, we observed significant main effects for both cued dimensions (spatial cue: $F(1, 30) = 20.24$, $p < 0.001$, $\eta^2_G = 0.047$, $BF_{10} > 100$; feature cue: $F(1, 30) = 8.56$, $p = 0.006$, $\eta^2_G = 0.012$, $BF_{10} = 2.05$; see Supplementary Figure S1). Participants responded faster following a valid spatial cue (M = 405 ms) compared to an invalid spatial cue (M = 441 ms). The same was true for the feature validity conditions (valid = 414 ms, invalid = 432 ms). Consistent with sensitivity and drift rate, we failed to detect a significant interaction between the two cue types, $F(1, 30) = 1.67$, $p = 0.21$, $\eta^2_G = 0.005$, $BF_{10} = 0.59$, further suggesting that the location and color cues independently influenced RT.

Experiment 2a

On average, participants responded fastest on SVFV trials (M = 481 ms) and slowest on SIFI trials (M = 522 ms). We observed a main effect of the spatial cue (valid: M = 484 ms, invalid: M = 511 ms), $F(1, 19) = 8.78$, $p = 0.008$, $\eta^2_G = 0.028$, $BF_{10} = 13.29$, but not of the feature cue (valid: M = 491 ms, invalid: M = 504 ms), $F(1, 19) = 2.28$, $p = 0.15$, $\eta^2_G = 0.006$, $BF_{10} = 0.57$, and no interaction, $F(1,19) = 1.04$, $p = 0.32$, $\eta^2_G = 0.003$, $BF_{10} = 0.45$. See Supplementary

Figure S2. This result is again consistent with the sensitivity and drift rate results presented in the main text.

Experiment 2b

We observed a marginal main effect of the spatial pre-cue, $F(1, 19) = 4.12$, $p = 0.057$, $\eta_G^2 = 0.006$, $B_{10} = 1.08$, and a significant effect of the feature pre-cues (valid: M = 484 ms, invalid: M = 515 ms), $F(1, 19) = 6.59$, $p = 0.019$, $\eta_G^2 = 0.017$, $B_{10} = 16.17$ (see Supplementary Figure S2). However, in contrast to the previous two experiments, here we also observed a significant interaction between cue types, $F(1, 19) = 16.82$, $p < 0.001$, $\eta_G^2 = 0.008$, $B_{10} = 2.25$. The spatial cueing effect was significant only on valid feature trials (SvFv: 465 ms vs. SiFv: 503 ms), $t(19) = 4.72$, $p < 0.001$, $d = 1.05$, $BF_{10} > 100$, but not on invalid feature trials (SvFi: 516 ms vs. SiFi: 513 ms), $t(19) = 0.25$, $p = 0.81$, $d = 0.056$, $BF_{10} = 0.24$. Similarly, the feature cueing effect was significant on valid spatial trials (SvFv vs. SvFi), $t(19) = 3.70$, $p = 0.0015$, $d = 0.83$, $BF_{10} = 25.42$, but not on invalid spatial trials (SiFv vs. SiFi), $t(19) = 0.82$, $p = 0.42$, $d = 0.18$, $BF_{10} = 0.31$. Interestingly, these analyses do not fully conform to the patterns observed within any of the DDM model outputs, as this is the only measure in which we detected an interaction.

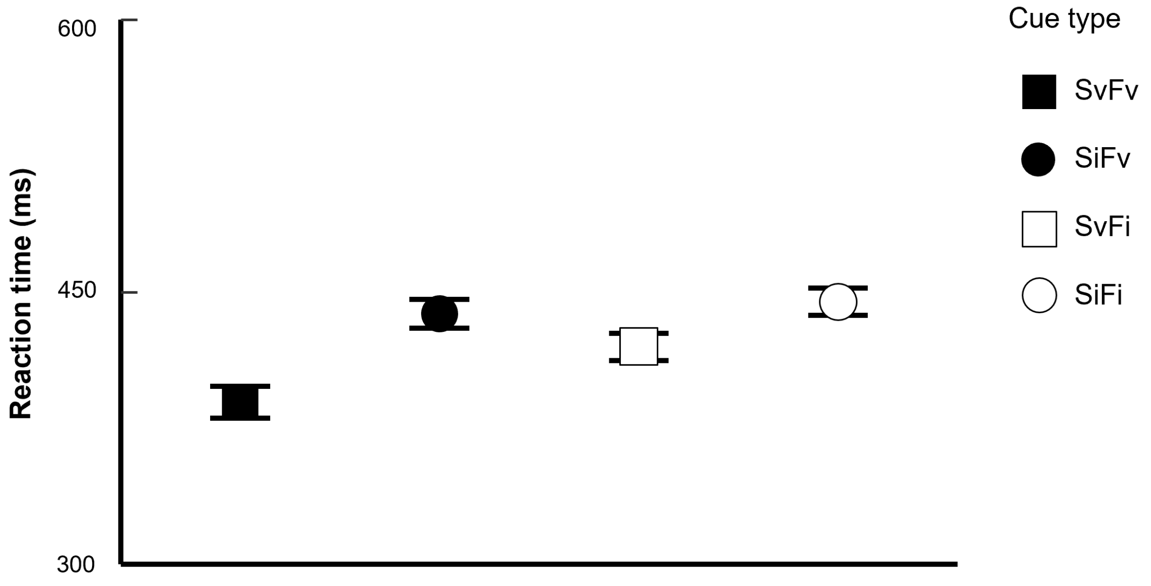

Supplementary Figure S1. Reaction times (RTs) from Experiment 1 are plotted with ±1 within-participant SEM error bars. Mean RTs were calculated for correct trials only.

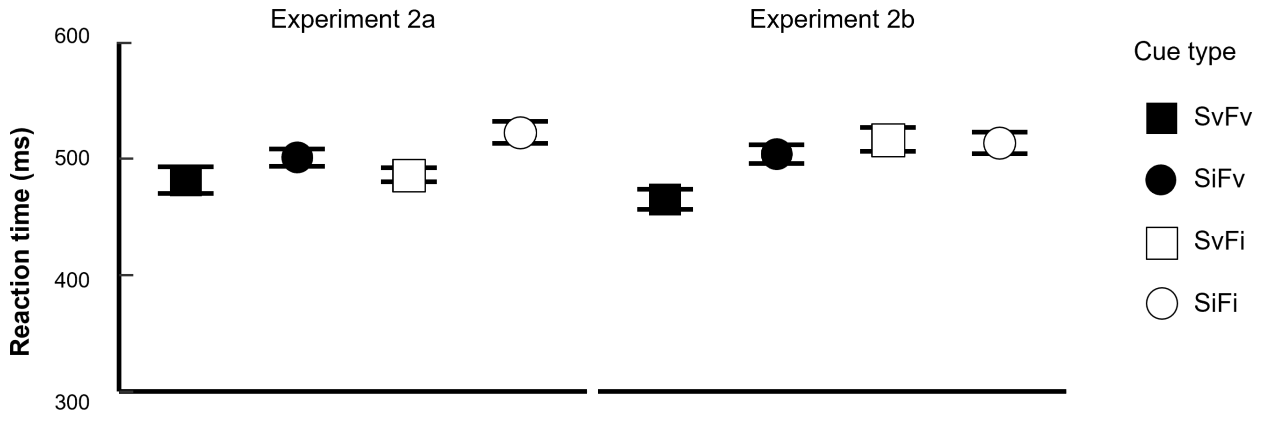

Supplementary Figure S2. Reaction times (RTs) from Experiments 2a (left panel) and 2b (right panel) are plotted with ±1 within-participant SEM error bars. Mean RTs were calculated for correct trials only.
